# Risk factors for dementia in the ninth decade of life and beyond: a study of the Lothian birth cohort 1921

**DOI:** 10.1186/s12888-017-1366-3

**Published:** 2017-06-02

**Authors:** Ruth A. Sibbett, Tom C. Russ, Ian J. Deary, John M. Starr

**Affiliations:** 1 Alzheimer Scotland Dementia Research Centre, The University of Edinburgh, Edinburgh, EH8 9JZ UK; 2 Centre for Cognitive Ageing and Cognitive Epidemiology, The University of Edinburgh, 7 George Square, Edinburgh, EH8 9JZ UK; 3 Department of Psychology, The University of Edinburgh, Edinburgh, UK; 4 Division of Psychiatry, The University of Edinburgh, Edinburgh, UK

**Keywords:** Dementia, Cohort, Incidence, Risk factor

## Abstract

**Background:**

With increasing numbers of people surviving beyond eighty years, this section of the population demands attention to reduce the impact of dementia. In order to develop effective preventative strategies, it is essential to understand age-specific risk factor profiles for dementia: do risk factors for dementia in those in their sixties and seventies persist into oldest age? The aims of this study were to determine incident dementia and to investigate the risk profile for dementia from age 79 to 95 years in a well-characterised cohort.

**Methods:**

Participants underwent intelligence testing at age 11 and were followed-up from at 79 years of age. Variables included: age, sex, age 11 IQ, *APOE* ɛ4, education, diabetes, hypertension, statin use, physical activity at leisure and in occupation, symptoms of depression, height, number of teeth, body mass index, blood pressure, cholesterol and HbA1c. Dementia cases were ascertained from death certificates, electronic patient records and clinical reviews. Logistic regression analysis determined the degree of risk for dementia associated with each variable. Analyses were completed both with and without the physical activity variables due to the significant number of missing data for these variables.

**Results:**

Of the eligible cohort, *n* = 410 participants remained dementia-free and *n* = 110 had developed probable dementia. When logistic regression analyses contained all variables, complete data was available for *n* = 234 (*n* = 48 with dementia). Results demonstrated that positive *APOE* ɛ4 carrier status (OR: 2.15, 95% CI: 1.04, 4.42) and greater lifetime physical activity (OR: 1.14, 95% CI: 1.02, 1.28) increased the risk for dementia. A reduction in risk for dementia was seen for hypertension (OR: 0.47, 95% CI: 0.23, 0.98). When physical activity variables were excluded, the number with complete data increased to *n* = 377 (*n* = 80 with dementia). *APOE* ɛ4 remained significant (OR: 2.37; 95% CI: 1.37, 4.07), as did hypertension (OR: 0.55; 95% CI: 0.32, 0.93).

**Conclusions:**

Dementia incidence was consistent with expected rates. The risk profile for dementia in this cohort of participants aged 79–95 confirmed previous findings that risk factors differ for those over 79 years. Further evidence is recommended in order that the risk profile for this age group can be accurately determined.

**Electronic supplementary material:**

The online version of this article (doi:10.1186/s12888-017-1366-3) contains supplementary material, which is available to authorized users.

## Background

Without clear means of prevention or cure, dementia is recognised to be one of the greatest public health challenges facing the ageing global population. Dementia rates are known to increase exponentially with age, from 5.5 per 1000 person-years in those aged 70–74, to 30.5 per 1000 person-years in those aged 80–84 [1]. With increasing numbers of people surviving into the ninth decade of life and beyond [2], this section of the population demands attention in order to reduce the impact of dementia [3]. Despite studies such as the 90+ Study [4]. (North America) and the Monzino 80-plus Study [5]. (Italy) the oldest in the population remain less well represented in dementia research.

In order to develop effective preventative strategies for dementia and ensure that these are directed appropriately, it is essential to identify potentially modifiable risk factors and understand whether these persist into oldest age. Significant modifiable risk factors for dementia demonstrated by replication within the literature include: diabetes [6], hypertension [7], hypercholesterolaemia [8], depression [9], smoking [10, 11], obesity [11]. and physical inactivity [11–13]. Previous studies have proposed that the risk factor profile for dementia changes with age, but the evidence is not conclusive [14, 15].

The present study draws on prospectively collected longitudinal data from the Lothian Birth Cohort 1921 (LBC1921). Participants were predominantly cognitively normal at baseline (aged 79 years) and underwent detailed follow-up to age 95 years. As a result, this study can add further evidence to the literature regarding risk factors for dementia in the oldest-old. Most participants in this cohort had also taken part in childhood intelligence testing at age 11 years. This is an unusual and valuable feature of the data for a study cohort of the oldest-old, given that lower childhood IQ has been shown to be a putative risk factor for dementia [16]. and is associated with several modifiable risk factors [17–19]. Dementia ascertainment had not previously been performed in LBC1921 and, although a number of participants would have developed dementia during the study period, there had not been any clear means of identifying all such participants. Mini-Mental State Examination (MMSE) [20]. was performed at each wave of follow-up and a small number were seen for clinical review following concerns raised regarding their cognitive function. Some participants self-reported a new diagnosis of dementia. This would have identified only a proportion of cases. There was no previous follow-up regarding dementia ascertainment for those who had died or left the study. Given the likelihood that participants with incident dementia were less likely to attend for follow-up, death records would be a valuable source data for dementia ascertainment, particularly where a diagnosis of dementia failed to be recorded in the secondary care records.

The primary aims of this study were: i) to determine cases of incident dementia within the LBC1921 study cohort from age 79–95, and ii) to investigate whether recognised modifiable risk factors for dementia (diabetes, hypertension, hypercholesterolaemia, depression, smoking, physical inactivity, obesity) remained risk factors for dementia in the ninth decade and beyond. These modifiable risk factors were considered together with key non-modifiable factors including; age 11 IQ, *APOE* ɛ4 status, and measures associated with socio-economic status.

The present study primarily drew on existing data for dementia ascertainment. Given the variability in methodology for using routinely collected data in the literature, we aimed to quantify the effectiveness of our dementia ascertainment method as a secondary outcome.

## Methods

### Study population

The LBC1921 is described in detail elsewhere [21], and is outlined briefly here. Almost all Scottish school pupils born in 1921 had their general intelligence tested at age ~ 11 years as part of the Scottish Mental Survey 1932 [22]. Beginning in 1999, the LBC1921 was designed in order to follow up some of the same participants in later life with the primary aim of investigating non-pathological cognitive ageing [23]. The LBC1921 includes 550 participants recruited from the Lothian area of Scotland, as relatively healthy, community-dwelling volunteers, most of whom had taken part in intelligence testing in 1932. Lothian is an area in southeast Scotland in which the largest settlement is the city of Edinburgh. Participants underwent the first wave of testing at approximately 79 years of age. Those participants surviving and continuing to consent to inclusion in the study were re-tested at regular intervals, at mean ages of about 83, 87, 90 and 92 years of age. The data were collected by questionnaire and one-to-one testing and included measures of socio-demographic, psychological, cognitive, medical, physiological, and genetic factors. Those participants self-reporting a history of dementia or scoring less than 24 on the MMSE at baseline were excluded from our study (*n* = 11), as were those who were missing baseline MMSE data (*n* = 2). Deaths were ascertained prospectively, with records for participants supplied by the General Registrar’s Office, Scotland [24]. Ethical approval was provided by the Lothian Research Ethics Committee (test waves 1–3) and the Scotland A Research Ethics Committee (test waves 4–5). Participants attending from wave 4 provided written consent for data linkage and access to health records.

### Dementia ascertainment

Surviving participants who continued to take part in the LBC1921 study were seen for routine follow-up as described previously. Follow-up for the purposes of detecting dementia diagnoses included the retrospective collection of evidence from the sources described below, from enrolment to age 95 years. Dementia cases were determined at a final consensus meeting on 15th December 2016. Death records for deceased participants were examined for evidence of cognitive impairment or dementia. Data from death records were collected from those available by 30th June 2016. For consenting participants, data were collected from medical and psychiatric electronic patient records for services in Lothian. Patients were located in the system using their Community Health Index (CHI) number, a unique number given to each patient within Scotland, recorded at every health service contact. Each hospital record accessed was read in full and examined for evidence of dementia or cognitive impairment since enrolment in the study. This included gathering both recorded confirmed diagnoses and evidence for diagnoses. Until 2014, general and psychiatric records were held on separate systems (Trak and PIMS respectively), but all records were subsequently incorporated into the Trak system. The final date for data collection from this source was 16th May 2016. For 26 participants, additional information was available as a result of clinical assessments undertaken by the authors (JMS or TCR) in the NHS or research setting. In the research setting, assessments were undertaken when impairment or decline was noted during the routine LBC1921 testing, or when a new diagnosis of dementia was self-reported. Data from these sources were collected until the consensus date.

Each case with evidence of cognitive impairment or dementia was considered at a consensus meeting (RAS, TCR, JMS) which included both a geriatrician and a psychiatrist. The meeting agreed upon whether the evidence supported a diagnosis of dementia and determined the subtype of dementia. Depending on the strength of the evidence, the diagnosis and subtype were deemed either ‘probable’ or ‘possible’. The criteria for probable and possible diagnoses utilised by the consensus are shown in Table 1 *.* Any disagreement on diagnosis was resolved through discussion.

**Table 1:** Consensus criteria for dementia case ascertainment

CONSENSUS CRITERIA FOR DEMENTIA CASE ASCERTAINMENT	
PROBABLE DEMENTIA	POSSIBLE DEMENTIA
ANY of the following (without opposing evidence from same/other source): - dementia diagnosis on death certificate (any part) - dementia diagnosed on clinical review (ICD-10/DSM-IV) - dementia diagnosis in electronic general medical records (Trak) - dementia diagnosis in electronic psychiatric records (PIMS) - ICD-10 criteria for dementia diagnosis met by data within any existing records	ANY of the following (without opposing evidence from same/other source): - recorded cognitive impairment on death certificate - cognitive impairment/decline recorded in notes, but incomplete evidence to meet ICD-10 diagnostic criteria - possibility of dementia recorded in notes but no formal diagnosis/incomplete evidence to meet ICD-10 diagnostic criteria

Dementia subtype diagnoses were made on a similar basis. Any dementia case with insufficient evidence to make a subtype diagnosis was classified as ‘unknown’ subtype. In order to minimise the risk of misclassification bias, probable dementia cases would be used as our primary outcome and possible cases would be excluded from the analyses. We would however repeat our analyses including possible dementia cases and include the results as supplementary information.

### Variables

Modifiable risk factors assessed in the present study were identified by matching those consistently reported in the literature (diabetes, hypertension, depression, hypercholesterolaemia, smoking, obesity, and physical inactivity) [12]. with data collected at LBC1921 test waves. We also included the following variables: age, sex, *APOE* ɛ4 status, age 11 IQ, number of teeth (as a post-retirement measure of socio-economic status [25]), height, and years in full-time, formal education. The full list of included variables is detailed in Additional file 1: Table S1.

Age at baseline was calculated according to the number of days between birth date and date attending wave 1 testing. The presence of at least one *APOE* ɛ4 allele was determined using genomic DNA isolated from venous blood [26]. Venous blood was also used to measure total serum cholesterol and HbA1c [21]. Any previous history of diabetes or hypertension, years in formal education, use of statins, and smoking status (previous, current or never) were self-reported by participants [26]. Body mass index (BMI) was calculated from height and weight, measured using a SECA stadiometer and digital SECA scales, respectively [27]. A trained research nurse measured sitting blood pressures (systolic and diastolic) using an Omron 705IT monitor [24]. Remaining teeth were counted during the general physical assessment [25]. Symptoms of depression were evaluated using the self-reported Hospital Anxiety and Depression Scale [28]. (HADS) at wave 1 [29]. Only the scores for the depression sub-scale were considered. Physical activity was self-reported by participants as part of a retrospective questionnaire at wave 2 follow-up (~age 83) [30]. Based on the methodology described by Hirvensalo and colleagues [31], responses were scored on a six-item scale according to increasing levels of physical activity. Responses predominantly related to leisure based activity: necessary movement, walking, walking/outdoor exercises, exercising until sweating, exercising several times per week, keep fit/heavy exercise. Participants indicated their perceived level of physical activity at three age ranges: 20–35, 40–55 and 60–75 years [30]. A lifetime score was calculated by the sum of the three scores. The physical effort required in a participant’s previous occupation was assessed using a single item [Q21] from the Job Content Questionnaire (JCQ) by Karasek [32, 33]. which was included, with permission, in the wave 2 questionnaire.

Age 11 IQ was derived from the results of the Moray House Test (MHT) no. 12, undertaken by participants in 1932 [26]. Following correction for age at testing, the cohort MHT scores were converted to IQ scores, with a standardised sample mean score of 100 and SD of 15 [26]. To demonstrate how the cohort IQ compares with the general population, we consider the raw MHT scores: 34.5 (SD: 15.5) was the mean score for Scotland, 37.3 (SD: 14.8) for those in Edinburgh schools, and 46.4 (SD: 12.1) for those recruited to LBC1921 [25].

### Statistical analyses

Statistical analysis was performed using IBM SPSS Statistics software version 21. The primary outcome of the study was the development of dementia. The analyses were completed for an outcome of probable dementia, with possible cases excluded. Univariate analysis was completed for each predictor variable, using either the Pearson chi-square or t-test. At this stage, a *p* value of <0.05 was used to demonstrate significant difference between those who developed dementia and those who did not. Binary logistic regression analysis was used to determine the risk for dementia associated with each predictor variable. For the purposes of logistic regression, the data for height and age 11 IQ were standardised so that a unit increase represented one standard deviation increase on the original scale. The following logistic regression models were completed using the ‘backward conditional’ function. The input for model 1 included all variables. The analyses for model 2 included all variables except lifetime physical activity and physical activity in occupation, which were excluded since data were missing for around one-third of participants (33.3 to 39.0% missing, with zero to 14.2% missing for all other variables). The analyses for models 1 and 2 were repeated to include both probable and possible dementia in the outcome, and the results are made available in the supplementary information.

## Results

Five hundred fifty participants recruited to the LBC1921 attended the first wave of data collection. We excluded 9 participants with an MMSE score of less than 24 at baseline, 2 participants missing MMSE results at baseline, 2 participants who self-reported a diagnosis of dementia at baseline and 10 participants with no follow-up data available. The eligible cohort (*n* = 527) included 305 (57.9%) females and 425 (80.6%) were known to be deceased by the 30th of June 2016. The mean age in years at wave 1 was 79.1 years (SD: 0.6). *APOE* ɛ4 carrier status was available for 521 participants (98.9%), with 139 (26.4%) recorded as carriers. The mean (standardised) age 11 IQ score was 100.1 (SD: 14.8), calculated from the 473 scores available (89.8% of the eligible cohort). The mean MMSE score for the eligible cohort was 28.3 (SD: 1.5). Descriptive statistics for those eligible for inclusion, and those excluded are shown in Table 2.

**Table 2:** Descriptive statistics for those included & excluded from the study

	Eligible cohort participants (*n* = 527)	Participants excluded from study (*n* = 23)
Deceased		
Living	102 (19.4%)	11 (47.8%)
Deceased	425 (80.6%)	12 (52.2%)
Sex		
Male	222 (42.1%)	12 (52.2%)
Female	305 (57.9%)	11 (47.8%)
Age at wave 1		
Mean age in years	79.1 (SD: 0.6)	79.2 (SD: 0.5)
*APOE* ɛ4 carrier status		
Carrier	139 (26.4%)	7 (30.4%)
Not carrier	382 (72.5%)	15 (65.2%)
Data missing	6 (1.1%)	1 (4.3%)
Age 11 IQ		
Data available	473 (89.8%)	20 (87.0%)
Data missing	54 (10.2%)	3 (14.3%)
Mean age 11 IQ	100.1 (SD: 14.8)	97.8 (SD: 19.6)
MMSE		
Data available	527 (100%)	21 (91.3%)
Data missing	-	2 (8.7%)
Mean MMSE	28.3 (SD: 1.5)	25.2 (SD: 3.3)

**Fig. 1:**
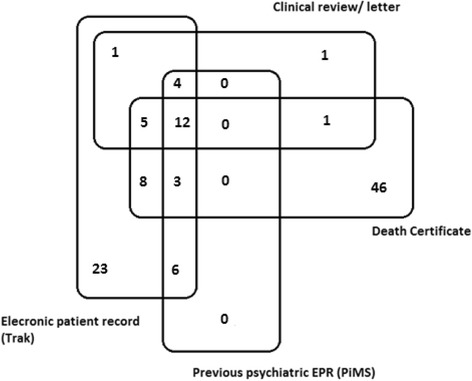
Number of probable dementia cases ascertained, by data source

One hundred twenty nine participants were found to have evidence of cognitive impairment or dementia in their records. A consensus diagnosis of probable dementia was agreed for 110 participants (38 probable Alzheimer disease, 25 probable vascular dementia, 9 probable mixed-type dementia, 1 probable progressive supra-nuclear palsy, 6 possible vascular dementia, 1 possible dementia in Parkinson’s disease, and 30 of unknown subtype) and a diagnosis of possible dementia was determined for 7 participants (1 possible vascular dementia, 6 unknown subtype). The remaining 12 cases considered had either insufficient information for diagnosis or evidence contradictory to a diagnosis of dementia (for example, the evidence supports a diagnosis of delirium rather than dementia). Figure 1 illustrates the number of probable cases ascertained by each data source, or combination of data sources. Almost two thirds of cases of probable dementia (63.6%) were determined based on a single source of information with the largest proportion of these single source diagnoses being based on death certificate data (Fig. 1).

All 7 cases of possible dementia were identified based on evidence from a single source (death certificate or electronic medical record). Of the 12 cases that did not meet the criteria for probable or possible dementia, 9 were determined based on a single data source, whilst the remaining 3 used two sources. The sources were as follows: 9 used evidence from the electronic medical records only, 1 used evidence from both the electronic medical records and the electronic psychiatric records and 2 used evidence from the electronic medical records and from clinical review.

Univariate analysis demonstrated significant differences between the group with probable dementia and the group without dementia for the following variables: positive *APOE* ɛ4 carrier status (*p* < 0.001), lower BMI at age 79 (*p* = 0.026) and current smoking status at age 79 (*p* = 0.039) (Table 3).

**Table 3:** Univariate analyses: comparisons between groups with and without probable dementia

Variable	No dementia (*n* = 410)	Probable dementia (*n* = 110)	Group comparison *p* value (chi-square or t-test)
Age at wave 1	*n = 410*	*n* = 110	
-mean age in years (SD)	79.1 (0.6)	79.0 (0.6)	0.610
Sex	*n = 410*	*n* = 110	
-% female	56.6	62.7	0.247
*APOE* ɛ4 carrier status^a^	*n = 404*	*n* = 110	
-% carrier *APOE* ɛ4	22.5	40.9	*<0.001*
Age 11 IQ (standardised)	*n = 365*	*n* = 102	
-mean score (SD)	100.0 (14.5)	100.1 (16.1)	0.948
Teeth	*n = 410*	*n* = 110	
-mean number of teeth (SD)	9.2 (9.4)	9.6 (8.9)	0.706
Height	*n = 409*	*n* = 107	
-mean height in cm (SD)	163.6 (9.4)	162.1 (9.2)	0.144
Formal education	*n = 409*	*n* = 109	
-mean number of years (SD)	10.9 (2.4)	11.0 (2.7)	0.732
History of diabetes	*n = 410*	*n* = 110	
-% positive history	5.4	4.5	0.731
HbA1c	*n = 356*	*n* = 98	
-mean HbA1c (SD)	5.7 (0.8)	5.7 (0.5)	0.703
History of hypertension	*n = 406*	*n* = 109	
-% positive history	42.1	34.9	0.171
Systolic blood pressure	*n = 408*	*n* = 109	
-mean BP in mmHg (SD)	168.9 (27.3)	166.0 (24.8)	0.315
Diastolic blood pressure	*n = 408*	*n* = 109	
-mean BP in mmHg (SD)	83.1 (13.2)	81.8 (12.3)	0.352
Statin use	*n = 347*	*n* = 99	
-% positive history	7.5	11.1	0.250
Total serum cholesterol	*n = 401*	*n* = 105	
-mean (SD)	5.7 (1.1)	5.6 (1.1)	0.891
Depression (HADS)	*n = 409*	*n* = 109	
-mean depression score (SD)	3.5 (2.3)	3.5 (2.4)	0.966
BMI	*n = 409*	*n* = 107	
-mean kg/m^2^ (SD)	26.4 (4.2)	25.4 (4.0)	*0.026*
Smoking status	*n = 410*	*n* = 109	
-% current smoker	8.5	2.8	*0.039*
Lifetime physical activity	*n* = 273	*n* = 74	
-mean total lifetime score (SD)	8.7 (3.1)	9.5 (3.0)	0.058
Physical effort required in occupation	*n = 248*	*n* = 69	
-mean score (SD)	2.1 (0.8)	2.3 (0.8)	0.065

^a^One or more alleles

Italicized results demonstrate significance of *p*<0.05

Following the exclusion of possible cases of dementia (*n* = 7), *n* = 520 participants were included in the logistic regression analyses, of which *n* = 110 had developed probable dementia. The results for these analyses are shown in Table 4. In both models the presence of an *APOE* ɛ4 allele increased the risk of dementia (model 2 OR: 2.37, 95% CI: 1.37, 4.07). A history of hypertension was associated with a decreased risk for dementia in both models (model 2 OR: 0.55, 95% CI: 0.32, 0.93). Increased height was associated with a decrease in risk for incident dementia in model 2 (model 2 OR: 0.73, 95% CI: 0.56, 0.96) and the same relationship approached significance in model 1 (model 1 OR: 0.71, 95% CI: 0.49, 1.01). A higher lifetime leisure-based physical activity score was associated with an increased risk of dementia in model 1 (OR: 1.14, 95% CI: 1.02, 1.28). Although current smoking was included in both models, the relationship with dementia did not reach significance. Age did not demonstrate an effect in any model, as might be expected in this narrow-age cohort.

**Table 4:** Logistic regression results

	Odds ratios (95% CI) for probable dementia	
	Model 1 (*n* = 234)	Model 2 (*n* = 377)
*APOE* ɛ4	2.15 (1.04,4.42)	2.37 (1.37,4.07)
Height (z score)	0.71 (0.49, 1.01)	0.73 (0.56, 0.96)
Hypertension	0.47 (0.23,0.98)	0.55 (0.32,0.93)
Current smoking	0.18 (0.02, 1.41)	0.25 (0.06, 1.09)
Lifetime physical activity	1.14 (1.02,1.28)	*-*

The variables entered into the analyses for each model were as follows: *Model 1-* age, sex, *APOE* ɛ4 carrier status, age 11 IQ (z score), number of teeth, height (z score), years in education, history of diabetes, HbA1c, history of hypertension, systolic blood pressure, diastolic blood pressure, cholesterol, use of statins, HADS depression score, BMI, smoking status, physical activity in occupation, lifetime physical activity (‘backward conditional’ method); *Model* 2- as model 1, but physical activity in occupation and lifetime physical activity excluded (‘backward conditional’ method)

To investigate the relationship with physical activity further, analysis for a third model was completed in which three individual age groups scores (20–35, 40–55, 60–75 years) replaced the lifetime physical activity score. All other variables were also included. Increased physical activity at age 20–35 years was significantly associated with incident dementia (OR: 1.35, 95% CI: 1.06, 1.73). The results of this model are shown in Additional file 2: Table S2.

Results for logistic regression analyses (models 1 and 2), repeated with possible cases included in the outcome, can be seen in Additional file 3: Table S3.

### Validation study

In order to validate our case ascertainment method using existing data sources, we completed a validation study comparing diagnoses extracted from existing data with diagnoses made on clinical review. Clinical reviews were performed for 26 participants. Of the 24 who were diagnosed as having dementia on clinical review, 23 had a diagnosis of dementia in at least one source of existing data. This would suggest that we would miss 4% of cases using existing data alone. Two participants seen for clinical review were not diagnosed as having dementia, but both had a diagnosis of dementia in the electronic medical records. This discrepancy might reflect the use of different diagnostic criteria, or the use of clinical judgement in clinical practice, particularly where evidence is ambiguous. Despite this discrepancy, our method would identify dementia in 88% of cases, with 4% being false negatives and 8% being false positives. Of the 17 cases identified as Alzheimer’s disease (AD) on clinical review, 14 (82%) had AD listed as a diagnosis within at least one data source. Of the 14 cases, 5 (36%) also had a different subtype diagnosis recorded in existing data, 7 (50%) also had dementia of an unspecified subtype recorded, while 2 (14%) cases listed only AD. Of the 2 cases identified as vascular dementia on clinical assessment, 1 had vascular dementia listed as a diagnosis within the existing data. Of the 3 cases identified as mixed Alzheimer’s and vascular dementia on clinical assessment 1 had a diagnosis of mixed dementia in the existing data. These findings demonstrate the usefulness of accessing records to find evidence that will support a subtype diagnosis based on recognised criteria. Our finding that overall dementia diagnoses were confirmed in 88% of cases is comparable, if not better than, validation procedures performed for other existing data sources or methodologies.

## Discussion

This study found that 21.2% of eligible, initially cognitively normal participants from the LBC1921 developed dementia from age 79 to 95 years. At the time of this study, 420 of 520 eligible participants had died, including 89 who had died with dementia. A total of 21 participants with dementia were alive at age 95. Our analyses indicated that the presence of an *APOE* ɛ4 allele and greater lifetime leisure-based physical activity increased the risk for dementia. A history of hypertension and increased height were found to reduce the risk for dementia.

The results of this study reinforce the importance of the *APOE* ɛ4 allele as a risk factor for the development of dementia [34, 35]. A number of studies have suggested a decline in the importance of *APOE* ɛ4 as a risk factor for dementia with advancing age [34, 36]. Somewhat to the contrary, our study has determined that *APOE* ɛ4 continues to be a significant risk factor for incident dementia from age 79 to 95.

Our results also indicated that a history of hypertension by age 79 was associated with a reduction in risk for dementia. This result supports the findings of previous studies that have demonstrated that the association of hypertension with dementia changes towards later life [37]. We might hypothesize that persons surviving and remaining dementia-free at the ninth decade of life, are no longer subject to any increased risk as a result of vascular factors such as hypertension. In simple terms, such risk factors have been used up and those with hypertension who were at the highest risk for dementia are more likely to have died from hypertension-related diseases prior to the onset of dementia. As a result, we might expect a paradoxical effect, much like that seen in this study. This hypothesis is supported by the direction of relationship for physical activity. Previous studies have hypothesized that a reduction in blood pressure is a consequence of the development of dementia and, although this mechanism is not fully understood, several processes have been proposed [37, 38]. Blood pressure may decline in early dementia due to the direct effect of neurodegeneration at the brainstem and hypothalamic nuclei- where arterial pressure is regulated- or it may be related to systemic changes such as weight loss, or any disease effecting the ability of the cardiovascular system to maintain perfusion pressures throughout the body [38]. Another possible explanation for the reduced risk is the potentially protective effect of antihypertensive agents, particularly as it is reported that antihypertensive use in hypertension is higher in older age [39–42].

The findings relating to physical activity were more unexpected with higher levels of overall leisure activity throughout adulthood being linked with an increased risk of developing dementia. As a consequence of missing data, the findings relating to physical activity were obtained for a smaller sample size and we must therefore be cautious in drawing inferences from these findings, particularly as they contradict studies that have previously indicated a link between midlife inactivity and dementia [11, 13]. The discrepancy between our findings and those of previous studies may be related to the method of data collection for these variables. Self-reporting physical activity levels throughout life at 79 years is likely to be subject to recall bias and variability between participants.

In this cohort, one standard deviation increase in height corresponded to 9.3 cm which was associated with an approximately 27% reduction in odds of possible or probable dementia (OR: 0.73, 95% CI: 0.56, 0.96). Our results are supported by the finding of a 2014 individual participant meta-analysis, that increasing height was related to a lower rates of death from dementia [43]. As concluded by the authors, since height is regarded as a marker of factors in early life, it may be these that are related to risk of dementia [43]. Like *APOE* ɛ4, we have demonstrated that decreased height continues to be a significant risk factor for dementia in oldest age. By demonstrating that certain recognised dementia risk factors are unchanged in oldest age, we can be more confident in our findings that the risk associated with other factors is changed in oldest age.

Contrary to much of the existing literature, no other factor considered in this study was found to be associated with dementia. We should consider however, that the prevalence of some conditions, including diabetes and depression, in our cohort was low and as a result, we were unlikely to detect anything except large effects, higher than those estimated by meta-analyses. [6, 9]. Power calculations determined that with binary logistic regression, setting alpha = 0.05 and the group sizes fixed at *n* = 410 (participants without dementia) and *n* = 110 (participants with dementia) with a base proportion of 5.4% (as for diabetes prevalence) in the *n* = 410, a minimum prevalence of 14.1% would be required in the *n* = 110 to detect a statistical difference with 80.0% power. Further investigation using case-control studies or much larger cohort studies are therefore required.

Moreover, given *n* = 110 people with dementia, the number of participants with each subtype of dementia was too few for analysis by individual subtype: combining cases of different aetiology may have affected the analysis. As previously noted, some of the data collected relied on recollection by the participant and was therefore subject to potential variability in reporting. The associations between our variables may also have affected our analyses. We attempted to minimise this as far as possible, but such bias could not be eliminated without excluding important variables. By examining many different possible predictors for dementia, in more than one model, there is also the potential for false positive findings. We limited the number of models in our analyses to two to reduce the chance of such false findings insofar as possible. A valuable strength of the study cohort is the presence of an intelligence test score from age 11 [16, 18, 21]. Each participant also underwent careful background assessment and thorough follow-up, providing a wealth of longitudinal data for the assessment of modifiable risk factors. The LBC1921 is a narrow-age cohort comprising ethnically, geographically and culturally homogenous participants, which means that we can rule out a number of potential confounding effects. Follow-up data were available for a satisfactory proportion of the original cohort to allow for analyses. The cohort demographics for those excluded from the analyses were similar to those included and it can therefore be assumed that the eligible cohort was a successful representation of the whole cohort. With a mean baseline MMSE of 28.1 (SD: 1.7) for those participants who subsequently developed dementia, we can be confident that we have identified truly incident, as opposed to prevalent, cases.

To assess the effectiveness of our dementia detection methodology, we sought to compare the incidence rate found against the rates determined by previous studies. Without knowing the age at diagnosis for a high proportion of dementia cases, the expected overall incidence over the study period had to be estimated (see Additional file 4). Had all cases of dementia been ascertained, we would have expected approximately 166 cases (see Additional file 5: Table S4). The 110 cases of dementia detected in this study therefore equates to 66.2% of the estimated number of cases arising over the same time period. This proportion is fairly consistent with a 2012 study of dementia diagnosis rates, which found that, within Lothian (the Health Board where the LBC1921 is resident), 68.3% of the expected cases of dementia had received a diagnosis [44]. We also sought to establish whether cases identified as possible dementia would be confirmed with additional follow-up. Of the 7 possible dementia cases, 5 were deceased at the time of the consensus meeting and no further follow-up could be completed. Electronic hospital records for the 2 other cases were accessed on 10th January 2017 and both contained evidence from that confirmed a formal diagnosis of dementia. It should be noted that neither case was seen for clinical review by ourselves and we did not therefore influence the diagnosis having been made.

This study has demonstrated the benefits of using multiple data sources for ascertainment. Our study returned the greatest number of cases from death certificates, which identified 68.2% of all cases of probable dementia, and 84.3% of all deceased participants with probable dementia. This finding would be in line with a previous Scottish study that found 71.5% of patients who die with dementia have the diagnosis on their death certificate [45]. Death certificates as a source of data benefit from their availability, but it is clear that the potential for missed cases remains. Many published UK studies utilising existing data for dementia ascertainment use only a single data source [46, 47]. As is the case with any dementia ascertainment procedure, the emphasis must be on achieving the most accurate representation of dementia incidence or prevalence within the population. Where possible, we would recommend that future studies consider inconsistencies between sources on a case-by-case basis. If there is reliable and consistent evidence in one source, the absence of a diagnosis in another source should not be assumed to equate to an absence of the disease. Where there is contradictory evidence, of similar weighting, from two or more sources, external evidence can be sought to clarify the diagnosis. This may take the form of a clinical review. Where no external evidence is available or possible, cases with contradictory evidence should be classified as possible cases and excluded from the analyses due to the risk of misclassification. Using existing data offers savings in terms of researcher and participant time and the associated financial costs. This method also allows for large population studies, where clinical diagnostic work-up is not feasible due to scale.

## Conclusions

In summary, the results of this study suggest that the presence of an *APOE* ɛ4 allele is a risk factor for incident dementia from age 79–95. A previous diagnosis of hypertension and increasing height were found to reduce the risk of incident dementia in the same age group. Increased leisure-based physical activity in adulthood was found to increase the risk for incident dementia, but including this variable in the analyses reduced the study sample size and we must therefore be cautious in drawing inferences from this finding, particularly as it contradicts previous studies. Our findings would support the hypothesis that the risk profile for dementia alters with age, however, further evidence would be required before the risk profile for the ninth decade of life and beyond could be accurately described.

## Additional files

Additional file 1: Table S1. LBC1921 Data Variables for Inclusion in Analyses. (DOCX 11 kb)

Additional file 2: Table S2. Logistic Regression Analysis with Physical Activity Age Groups. (DOCX 13 kb)

Additional file 3: Table S3. Logistic Regression Analyses for Probable and Possible Dementia. (DOCX 14 kb)

Additional file 4: Estimated Dementia Incidence. (DOCX 15 kb)

Additional file 5 **Table S4.** Estimated Incidence of Dementia in LBC1921. (DOCX 15 kb)

## Abbreviations

APOE: Apolipoprotein E
BMI: Body mass index
CHI: Community health index
HADS: Hospital anxiety and depression scale
LBC1921: Lothian Birth Cohort 1921
MHT: Moray house test
MMSE: Mini-mental state examination
NHS: National health service
PIMS: Patient information management system
Trak: TrakCare
