# Supplementary material for: Risk factors for dementia in the ninth decade of life and beyond: a study of the Lothian birth cohort 1921
Source: BMC Psychiatry. 2017 Jun 2;17:205. doi: 10.1186/s12888-017-1366-3 (PMC5455126; doi:10.1186/s12888-017-1366-3)
Supplement: Supplementary file 1 — LBC1921 Data Variables for Inclusion in Analyses. (DOCX 11 kb) [file 12888_2017_1366_MOESM1_ESM.docx]

*Additional file 1: Table S1. LBC1921 data variables for inclusion in analyses*

| **LBC 1921 Data Elements Selected for Inclusion** |
| --- |
| Age- *from birth to wave 1 testing*  Sex  Age 11 IQ score (standardised)  Interview/ questionnaire measures:  Years in full-time, formal education- *self reported at wave 1*  Self-reported history of diabetes- *at wave 1*  Self-reported history of hypertension- *at wave 1*  Statin use- *at wave 1*  Self-reported smoking status- *at wave 1*  Physical activity at age 20-35, 40-55 and 60-75- *self-reported at wave 2*  Physical effort required in occupation- *self-reported at wave 2*  Symptoms of depression (HADS depression score)- *at wave 1*  Physical measures:  Number of teeth remaining- *at wave 1*  Height- *at wave 1*  Body mass index (BMI)- *at wave 1*  Sitting systolic & diastolic blood pressure- *at wave 1*  Blood measures:  Total serum cholesterol- *at wave 1*  HbA1c- *at wave 1*  APOE e4 carrier status |
