# Supplementary material for: Risk factors for dementia in the ninth decade of life and beyond: a study of the Lothian birth cohort 1921
Source: BMC Psychiatry. 2017 Jun 2;17:205. doi: 10.1186/s12888-017-1366-3 (PMC5455126; doi:10.1186/s12888-017-1366-3)
Supplement: Supplementary file 2 — Logistic Regression Analysis with Physical Activity Age Groups. (DOCX 13 kb) [file 12888_2017_1366_MOESM2_ESM.docx]

*Additional file 2: Table S2. Logistic Regression Analysis with Physical Activity Age Groups*

|  | **Odds Ratios (95% CI) for Probable Dementia** |
| --- | --- |
|  | **Model 3 (n=234)** |
| ***APOE* ɛ4** | 2.20 (1.07. 4.53) |
| **Height (z score)** | 0.69 (0.48, 1.00) |
| **Hypertension** | 0.46 (0.22, 0.96) |
| **Current smoking** | 0.16 (0.02, 1.29) |
| **Physical activity, age 20-35 years** | 1.35 (1.06, 1.73) |

*Note.* The variables entered into the analyses were as follows: age, sex, *APOE* ɛ4 carrier status, age 11 IQ (z score), number of teeth, height (z score), years in education, history of diabetes, HbA1c, history of hypertension, systolic blood pressure, diastolic blood pressure, cholesterol, use of statins, HADS depression score, BMI, smoking status, physical activity in occupation, physical activity at age 20-35, physical activity at age 40-55, physical activity at age 60-75 (‘backward conditional’ method).
