# Supplementary material for: Risk factors for dementia in the ninth decade of life and beyond: a study of the Lothian birth cohort 1921
Source: BMC Psychiatry. 2017 Jun 2;17:205. doi: 10.1186/s12888-017-1366-3 (PMC5455126; doi:10.1186/s12888-017-1366-3)
Supplement: Supplementary file 3 — Logistic Regression Analyses for Probable and Possible Dementia. (DOCX 14 kb) [file 12888_2017_1366_MOESM3_ESM.docx]

*Additional file 3: Table S3. Logistic Regression Analyses for Probable and Possible Dementia*

|  | **Odds Ratios (95% CI) for Probable and Possible Dementia** | |
| --- | --- | --- |
|  | **Model 1 (n=237)** | **Model 2 (n=382)** |
| ***APOE* ε4** |  | **2.24 (1.32,3.78)** |
| **Height*** |  | **0.74 (0.57,0.96)** |
| **Education** | **0.86 (0.74,1.00)** |  |
| **Hypertension** | 0.57 (0.29,1.12) |  |
| **BMI** |  | 0.95 (0.89,1.01) |
| **Current smoker** | 0.31 (0.07,1.43) | 0.37 (0.11,1.26) |
| **Lifetime physical activity** | **1.13 (1.01,1.25)** | - |

*Note.* The variables entered into the analyses for each model were as follows: *Model 1-* age, sex, *APOE* ɛ4 carrier status, age 11 IQ (z score), number of teeth, height (z score), years in education, history of diabetes, HbA1c, history of hypertension, systolic blood pressure, diastolic blood pressure, cholesterol, use of statins, HADS depression score, BMI, smoking status, physical activity in occupation, lifetime physical activity (‘backward conditional’ method); *Model* 2- as model 1, but physical activity in occupation and lifetime physical activity excluded (‘backward conditional’ method).
