# Supplementary material for: Risk factors for dementia in the ninth decade of life and beyond: a study of the Lothian birth cohort 1921
Source: BMC Psychiatry. 2017 Jun 2;17:205. doi: 10.1186/s12888-017-1366-3 (PMC5455126; doi:10.1186/s12888-017-1366-3)
Supplement: Supplementary file 4 — Estimated Dementia Incidence. (DOCX 15 kb) [file 12888_2017_1366_MOESM4_ESM.docx]

*Additional file 4*

**Estimated dementia incidence**

A European meta-analysis described annual incidence rates of dementia of 1.6% in those aged 75-79 years, 3.1% in those aged 80-84 years, 4.9% in those aged 85-89 and 7% in those aged 90 and over.[1] In order to formulate the estimate, our calculations needed to take into consideration the number of participants who died in the preceding year, and the cohort size adjusted accordingly. Participants with a diagnosis of dementia at death were not counted as ‘deaths’ as expected cases had already been excluded from calculations for the following year as a result of a positive diagnosis. Taking the above values as the expected annual incidence rates, we would expect that of our eligible cohort of 520 participants (excluding those identified as possible dementia), 169 participants would have developed dementia by the conclusion of our study (*additional table 3*).

The 110 cases of dementia ascertained in this study therefore equates to 66.2% of the estimated number of cases arising over the same time period. Given that our ascertainment method primarily relied on diagnosed cases of dementia, it is useful to consider the proportion detected in the context of diagnostic rates for the region. A 2012 study of dementia prevalence and diagnosis rates found that within Lothian 68.3% of the expected cases of dementia had received a diagnosis.[2] We can be confident in our assumption that not all cases of dementia are diagnosed in the community, as in our cohort, cases of previously undiagnosed dementia were identified on clinical review, following concerns raised at routine follow-up. Overall, it can be seen that the number of cases of dementia ascertained in this study corresponded fairly closely to the predicted number of diagnosed cases for the same cohort. With the number of cases detected falling below the total estimate of 166.1, it is unlikely that our ascertainment method has falsely identified any participants without dementia, as having dementia. We expected incidence to be lower in our cohort, than the rates described for the general population, as a result of higher IQ and generally good health at baseline. Conversely, these participants are motivated to take part in research and therefore may be more likely to be motivated to access health services. Further to this, undergoing regular cognitive testing may have highlighted any issues with memory that might not have been otherwise noted.

**References**

1. Fratiglioni L, Launer LJ, Andersen K, Breteler MM, Copeland JR, Dartigues JF. **Incidence of dementia and major subtypes in Europe: A collaborative study of population based cohorts.** Neurologic Diseases in the Elderly Research Group. *Neurology* 2000; **54** (11 Suppl. 5): S10-S15.
2. Alzheimer's Society. **Mapping the Dementia Gap 2012: Progress on improving diagnosis of dementia 2011-2012.** <http://www.healthcare-today.co.uk/doclibrary/documents/pdf/826_Mapping_the_dementia_gap.pdf>. Accessed 9 August 2016.
