# Supplementary material for: Risk factors for dementia in the ninth decade of life and beyond: a study of the Lothian birth cohort 1921
Source: BMC Psychiatry. 2017 Jun 2;17:205. doi: 10.1186/s12888-017-1366-3 (PMC5455126; doi:10.1186/s12888-017-1366-3)
Supplement: Supplementary file 5 — Table S4. Estimated Incidence of Dementia in LBC1921. (DOCX 15 kb) [file 12888_2017_1366_MOESM5_ESM.docx]

*Additional file 5: Table S4. Estimated incidence of dementia for LBC 1921*

| **Age** (years) | **Number disease free after previous year** | **Number deceased in the previous year** | **Sample size at age**  (living, dementia-free) | **Expected annual incidence**  (EURODEM) | **Estimated number of cases expected** |
| --- | --- | --- | --- | --- | --- |
| 79 | 520 | 1 | 519 | 1.6% | 8.3 |
| 80 | 510.7 | 4 | 506.7 | 3.1% | 15.7 |
| 81 | 491 | 20 | 471 | 3.1% | 14.6 |
| 82 | 456.4 | 22 | 434.4 | 3.1% | 13.5 |
| 83 | 420.9 | 17 | 403.9 | 3.1% | 12.5 |
| 84 | 391.4 | 26 | 365.4 | 3.1% | 11.3 |
| 85 | 354.1 | 28 | 326.1 | 4.9% | 16.0 |
| 86 | 310.1 | 24 | 286.1 | 4.9% | 14.0 |
| 87 | 272.1 | 28 | 244.1 | 4.9% | 12.0 |
| 88 | 232.1 | 22 | 210.1 | 4.9% | 10.3 |
| 89 | 199.8 | 33 | 166.8 | 4.9% | 8.2 |
| 90 | 158.6 | 20 | 138.6 | 7% | 9.7 |
| 91 | 128.9 | 25 | 103.9 | 7% | 7.3 |
| 92 | 96.6 | 20 | 76.6 | 7% | 5.4 |
| 93 | 71.2 | 17 | 54.2 | 7% | 3.8 |
| 94 | 50.4 | 15 | 35.4 | 7% | 2.5 |
| 95 | 32.9 | 4 | 28.9 | 7% | 2  (*deduct 1) |
|  |  |  |  |  | **Total= 166.1** |

*(*Less than half of the year studied, therefore 1 expected case from 2 was deducted from total)*
